# Nerve Growth Factor in Diabetes Mellitus: Pathophysiological Mechanisms, Biomarkers and Therapeutic Opportunities

**DOI:** 10.3390/ph18121805

**Published:** 2025-11-26

**Authors:** Mattia Massimino, Mariangela Rubino, Maria Resilde Natale, Luca Salerno, Stefania Belviso, Elettra Mancuso, Annamaria Dagostino, Davide Demasi, Flora Barreca, Carolina Averta, Angela Palummo, Gaia Chiara Mannino, Francesco Andreozzi

**Affiliations:** 1 Department of Medical and Surgical Science, University Magna Graecia of Catanzaro, Viale Europa, 88100 Catanzaro, Italy; mattia.massimino@hotmail.it (M.M.); mariangela.rubino@unicz.it (M.R.); resilde94@gmail.com (M.R.N.); l.salerno@unicz.it (L.S.); annamaria.dagostino94@gmail.com (A.D.); ddms.dd@gmail.com (D.D.); florabarreca@outlook.it (F.B.); angela.palummo@gmail.com (A.P.); andreozzif@unicz.it (F.A.); 2 Azienda Ospedaliera Universitaria “Renato Dulbecco” University Hospital of Catanzaro, 88100 Catanzaro, Italy; stefania.belviso@live.it; 3 Department of Science of Health, University Magna Graecia of Catanzaro, Viale Europa, 88100 Catanzaro, Italy; elettramancuso@unicz.it; 4 Department of Experimental and Clinical Medicine, University Magna Graecia of Catanzaro, Viale Europa, 88100 Catanzaro, Italy; carolinaaverta90@gmail.com

**Keywords:** type 2 diabetes mellitus, nerve growth factor, proNGF, biomarkers, therapeutic targets, diabetes complications, translational medicine

## Abstract

**Background**: Type 2 diabetes mellitus represents a global health challenge, with chronic hyperglycemia leading to a spectrum of microvascular and macrovascular complications. This narrative review provides a comprehensive and integrated analysis of the nerve growth factor (NGF) axis as a key, yet underrecognized, pathogenic mechanism. **Methods**: This narrative review was conducted in accordance with scholarly standards for non-systematic syntheses (SANRA). We included both clinical and preclinical studies focusing on NGF/proNGF biology and interventions across major diabetes complications. **Discussion**: Growing evidence highlights NGF as a pivotal mediator at the crossroads of neuronal, vascular and metabolic pathways. In diabetes, a disrupted balance between mature NGF and its precursor proNGF, favors the detrimental p75NTR pathway, leading to increased cellular stress, inflammation and apoptosis. In this narrative review, we examine how a decline in mature NGF and a relative excess of proNGF contribute to the pathophysiology of diabetic complications across various organ systems. We highlight the dual role of the NGF axis: while NGF-TrkA signaling consistently confers neuroprotective and vasculoprotective benefits, unchecked proNGF-p75NTR activity amplifies tissue damage. **Conclusions**: Collectively, the evidence identifies NGF as a candidate biomarker for both early tissue distress and therapeutic monitoring. We conclude by outlining key priorities for future research, including the development of standardized assays and the initiation of well-designed clinical trials to translate these promising strategies for early detection and treatment of diabetes-related complications.

## 1. Introduction

Type 2 diabetes mellitus (T2DM) is a chronic metabolic disorder characterized by hyperglycemia resulting from inadequate insulin secretion or action. According to the International Diabetes Federation (IDF), as of 2024, T2DM has reached pandemic proportions, affecting an estimated 588 million adults worldwide, a figure projected to rise to 852 million by 2050. Chronic hyperglycemia in T2DM leads to progressive tissue damage and a broad spectrum of complications that substantially contribute to global morbidity and mortality [1]. These complications are traditionally classified as microvascular, including diabetic neuropathy, retinopathy, nephropathy and cognitive impairment, and macrovascular, such as cardiovascular, cerebrovascular and peripheral arterial disease [2]. Although heterogeneous in clinical presentation, these complications share a common pathophysiological substrate driven by persistent hyperglycemia-induced oxidative stress, low-grade inflammation and vascular dysfunction [3]. These complications not only reduce quality of life but also impose a significant burden on healthcare systems globally, contributing to increased hospitalization, disability and economic costs [4]. Given their substantial impact on health systems and individual well-being, the identification of early biomarkers and novel therapeutic targets is imperative.

**Figure 1:**
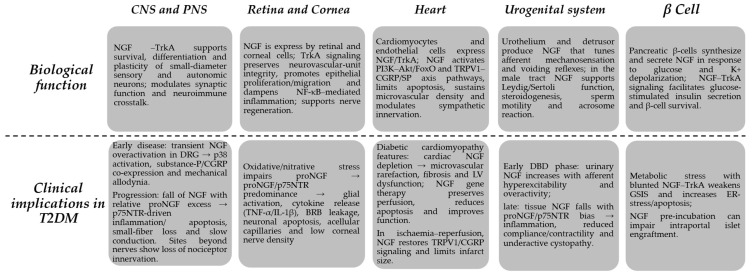
Biological and pathophysiological roles of NGF in diabetes. Under physiological conditions, NGF supports survival and function of sensory and autonomic neurons, maintains corneal and cutaneous integrity, promotes angiogenic and endothelial homeostasis, and contributes to β-cell function and balanced immune responses. In diabetes, dysregulation of the NGF/proNGF axis, characterized by altered NGF availability and increased p75NTR, shifts these protective actions towards nociceptor sensitization, neurodegeneration, impaired wound and corneal healing, microvascular damage and dysfunction of the lower urinary tract and reproductive system. → direction of effect/sequence.

Recent literature highlights nerve growth factor (NGF) as a key neurotrophin potentially serving as a promising diagnostic biomarker for Alzheimer’s disease [5], frontotemporal dementia [6], malignancies [7] and overactive bladder syndrome [8]. It is also increasingly regarded as a feasible therapeutic target for rheumatoid arthritis [9], osteoarthritis [10], neurotrophic keratitis [11], dry-eye disease [12], retinitis pigmentosa [13], Alzheimer’s disease [14], pediatric traumatic brain injury (TBI) [15] and bone metastasis-related pain [16], thereby opening new avenues to improve diagnostic precision, enable timely interventions and reduce long-term disease burden [17]. NGF mediates its biological effects through two major receptor pathways with opposing functions. The high-affinity tropomyosin receptor kinase A (TrkA) facilitates neuronal differentiation, growth and survival, while the low-affinity p75 neurotrophin receptor (p75NTR) promotes apoptotic and stress-related responses, particularly in complex with sortilin. NGF is initially synthesized as a precursor molecule (proNGF), which must undergo proteolytic cleavage to generate its mature, functionally active form. While mature NGF predominantly binds TrkA to exert neuroprotective effects, proNGF preferentially binds p75NTR [18]. In diabetes, the equilibrium between mature NGF and proNGF is frequently disrupted, with a relative predominance of proNGF. This altered NGF/proNGF ratio favors p75NTR-mediated apoptotic pathways, thereby contributing to the pathophysiology of diabetes-related complications by exacerbating cellular stress, promoting apoptosis and impairing tissue repair mechanisms [19] (Figure 1). The primary objective of this narrative review is to consolidate current mechanistic and preclinical insights into NGF/proNGF in diabetes-related complications and to define translational and clinical research priorities that can clarify the clinical relevance of NGF/proNGF as both a biomarker and a therapeutic target in managing diabetic neuropathy, retinopathy, cardiomyopathy, urogenital dysfunction and β cell function.

## 2. Materials and Methods

This work was conducted as a narrative review, following scholarly standards for non-systematic literature syntheses (SANRA) [20]. The purpose was to provide an updated and comprehensive overview of the role of NGF and proNGF in diabetes mellitus and its complications, integrating evidence from experimental and clinical studies.

### 2.1. Search Strategy and Sources

A literature search was performed in PubMed/MEDLINE, Scopus and the Cochrane Central Register of Controlled Trials (CENTRAL). The time window considered extended from 1977 to October 2025, in order to include both historical and more recent studies relevant to NGF biology in diabetes. The following combinations of keywords and Medical Subject Headings (MeSH) were used in various iterations: “nerve growth factor” OR “NGF” OR “proNGF” OR “neurotrophins” AND “diabetes” OR “diabetes mellitus” OR “type 1 diabetes” OR “type 2 diabetes” OR “diabetic complications”, together with additional terms related to specific clinical domains (neuropathy, retinopathy, keratopathy, nephropathy, encephalopathy, cardiomyopathy, urogenital dysfunction and β-cell function). Searches were restricted to articles published in English. Reference lists of key articles and relevant reviews were also screened to identify additional pertinent sources that might not have emerged directly from database queries.

### 2.2. Study Selection and Eligibility

As customary in narrative reviews, selection was not based on a rigid stepwise screening process, but rather on consistency with the thematic scope and clinical–biological relevance. However, explicit inclusion and exclusion criteria guided the evaluation of the literature.

### 2.3. Inclusion and Exclusion Criteria Included

Original research articles (in vivo, in vitro, ex vivo or clinical), observational studies, randomized or non-randomized trials and translational investigations; studies addressing NGF, proNGF, their receptors or related downstream pathways and treatment in the context of diabetes mellitus or its complications; articles published in English within the predefined time frame (1977–2025).

Studies unrelated to diabetes or NGF biology; articles not written in English; case reports without mechanistic relevance, conference abstracts, editorials, letters and duplicate publications; interventional studies targeting unrelated molecular pathways without reference to NGF or proNGF.

Study quality and potential sources of bias (e.g., sample size, risk of confounding, lack of masking, incomplete outcome reporting) were assessed qualitatively and explicitly considered when interpreting mechanistic coherence and translational relevance, without applying a formal numerical scoring system.

Applying these criteria, a total of 68 studies were selected for detailed synthesis, including 56 preclinical (in vitro/in vivo/ex vivo) investigations and 12 clinical studies in humans. When uncertainty arose, full texts were retrieved and assessed for relevance. A broad range of study types was retained to capture the multifaceted role of NGF and proNGF in diabetes pathophysiology and potential therapeutic targeting. Findings were grouped narratively to highlight converging themes, emerging therapeutic approaches and gaps in knowledge.

## 3. Discussion

### 3.1. Diabetic Neuropathy

Diabetic neuropathy (DN) comprises a diverse group of clinical syndromes resulting from diabetes-induced damage to the peripheral nervous system. Among these, diabetic peripheral neuropathy (DPN) is the most frequently encountered form, affecting approximately 50% of patients with T2DM over a ten-year period [21]. DPN constitutes a major complication of diabetes, as it substantially impairs quality of life and is a significant contributor to both morbidity and mortality [22]. DPN is characterized by a biphasic course, comprising an early metabolic phase, that is potentially reversible and a late structural phase, associated with irreversible neuronal damage. The metabolic phase involves functional disturbances such as altered nerve conduction and increased sensitivity, reflecting compensatory mechanisms like axonal regeneration, remyelination and synaptogenesis mediated by neurotrophic signaling. In contrast, the structural phase is marked by sensory deficits including thermal hypoalgesia and mechanical allodynia, attributed to reduced neurotrophic support and progressive neuronal degeneration [23,24].

The pathogenesis of DN is multifactorial, involving chronic hyperglycemia, in addition to biochemical and vascular abnormalities. Key mechanisms include activation of the polyol pathway, accumulation of advanced glycation end products (AGEs), impaired microvascular perfusion, inflammation, oxidative stress, autoimmune responses and reduced availability of neurotrophic factors [23].

Nociceptive neurons in the dorsal root ganglia (DRG), which give rise to Aδ and C fibers, are subdivided into peptidergic neurons, expressing neuropeptides such as substance P (SP) and calcitonin gene-related peptide (CGRP), and dependent on NGF-TrkA signaling and non-peptidergic neurons that rely primarily on Glial cell line-Derived Neurotrophic Factor (GDNF) mediated trophic support [25].

NGF is essential for the development, differentiation and long-term maintenance of neuronal populations in both the central and peripheral nervous systems; its trophic actions are most prominent in small-caliber sensory and autonomic fibers, a feature particularly relevant in the context of DN [23].

Across experimental diabetes, NGF levels are context-dependent and these differences matter because they align with the time point, tissue compartment, animal strain/model, and concomitant interventions assessed. Studies that detect higher NGF typically capture early windows of nociceptive sensitization. In streptozotocin (STZ) rats soon after diabetes induction, DRG and spinal cord show a transient up-shift in NGF pathway activity, with increased TrkA/p75 expression and kinase activation accompanying mechanical hyperalgesia; electroacupuncture delivered in this early window reduced NGF content in DRG and spinal cord, lowered TrkA phosphorylation and p75NTR in skin and improved nociceptive thresholds, consistent with the interpretation that local NGF overactivation contributes to early pain behaviors [24].

**Figure 2:**
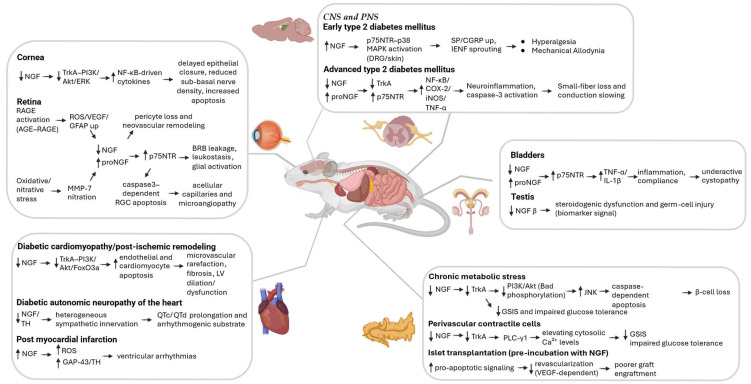
Schematic representation of NGF/proNGF signaling pathways and target organs in preclinical model of DM. Symbols: ↑, increased levels/expression; ↓, decreased levels/expression; → direction of effect/sequence. Modify from BioRender. Mannino, G. (2025) https://BioRender.com/dwsteip.

In *db/db* mice, NGF and substance P (SP) are elevated at 5–8 weeks, coinciding with mechanical allodynia; they normalize by 12 weeks and fall below control by 16 weeks; mechanistically, the same group linked early allodynia to NGF-p38 signaling, since anti-NGF reduced DRG p38 phosphorylation and attenuated allodynia [25] (Figure 2).

An STZ study that profiled redox and behavioral outcomes reported an NGF surplus in DRG early after induction; carvedilol lowered DRG NGF, reduced malondialdehyde, restored glutathione/SOD activities and improved pain behaviors [26].

Consistent with these observations, targeted interference with NGF–p38 signaling in *db/db* mice reduces mechanical allodynia and lowers p38 phosphorylation in lumbar DRG, alongside down-regulation of iNOS, COX-2 and TNF-α implicating NGF-driven p38 as a key driver of early nociceptive remodeling; notably, DRG p38 activation peaks at 5–10 weeks and returns toward control by 12 weeks [27].

Complementing this DRG-centric mechanism, during the same allodynic window the cutaneous compartment shows a transient increase in intraepidermal nerve fiber density, specifically PGP9.5^+^ and TrkA^+^ peptidergic fibers, which is reversed by systemic anti-NGF and by intrathecal p38 inhibition and normalizes by 16 weeks. This indicates compartment- and stage-specific dynamics, and DRG sensitization coexisting with transient cutaneous hyperinnervation [28].

Gao et al. document declining NGF with progression, particularly in peripheral nerves and DRG, aligning with trophic failure and structural loss. NGF levels fall first in DRG and later in dorsal horn; the persistent DRG deficit paralleled sustained hyperalgesia, whereas dorsal-horn NGF partially recovered as allodynia waned. Exogenous mNGF raised NGF levels in both compartments and partially normalized mechanical thresholds, supporting a rescue effect once endogenous NGF becomes insufficient [29].

Multiple STZ and non-obese diabetic mouse studies found reduced NGF mRNA/protein in sciatic nerve or its roots, with reversals under specific interventions. Endurance training for six weeks increased NGF transcripts in sensory and motor spinal segments, whereas treadmill paradigms in other preparations did not change sciatic-nerve NGF/BDNF protein and even increased 4-HNE, underscoring how exercise mode, intensity and readout determine observed effects [30,31].

Beyond descriptive pathophysiology, converging preclinical data show that context-aware augmentation of the NGF milieu can ameliorate diabetic nerve dysfunction.

In bone-marrow sensory fibers, a locus increasingly recognized as neuropathy-sensitive, STZ diabetes down-regulated nociceptor innervation; NGF gene therapy increased PGP 9.5+ and SP+ fiber densities, corrected nociceptor-mediated stem-cell mobilization after ischemic injury and improved healing, expanding the territory of NGF insufficiency beyond classical nerve targets [32].

Human evidence, though still limited, leans toward a circulating deficit in established DPN. Cross-sectional studies report lower serum NGF in patients with painful or moderate–severe DPN, often inversely correlated with neuropathy scores and with higher malondialdehyde, suggesting convergence between oxidative stress and neurotrophic insufficiency [33].

In STZ-diabetic rats subjected to sciatic crush, a heparin–poloxamer thermosensitive hydrogel co-delivering NGF and bFGF provided sustained release, enhanced Schwann-cell proliferation, promoted axonal regeneration and remyelination, improved motor recovery, and engaged PI3K/Akt, JAK/STAT3 and MAPK/ERK signaling outperforming bolus growth-factor administration or vehicle, and illustrating that controlled local NGF exposure can overcome a hostile diabetic microenvironment [34].

In STZ-diabetic nude mice, intramuscular transplantation of cryopreserved human dental-pulp stem cells improved sensory and motor nerve-conduction velocities and sciatic-nerve blood flow; transplanted cells expressed human NGF and VEGF near the grafts and neutralizing antibodies against either factor negated the conduction benefit, highlighting a paracrine NGF/VEGF mechanism [35].

Donor biology constrains trophic provisioning. Bone-marrow mononuclear cells from young, non-diabetic donors improved thermal sensitivity, sciatic blood flow and conduction, whereas cells from adult or diabetic donors did not have an effect that paralleled lower NGF and bFGF transcript levels in the ineffective donor cells [36].

At the glial-niche level, metformin countered hypoxia-induced Schwann-cell injury via AMPK, reducing apoptosis and increasing the expression and secretion of NGF, BDNF, GDNF, and N-CAM; these effects were suppressed by the AMPK inhibitor compound C, indicating pathway dependence [37].

Botanicals can also up-regulate neurotrophins.

*Gymnema sylvestre* reversed sciatic-nerve NGF loss, improved axonal architecture and attenuated inflammation/oxidative stress in STZ rats, while taurine in STZ rats restored spinal NGF and increased phosphorylated TrkA, Akt and mTOR [38]; in a complementary STZ-diabetic rat model, taurine restored spinal cord NGF expression and increased the activating phosphorylation of TrkA, Akt and mTOR; importantly, NGF neutralization or Akt/mTOR inhibition largely blunted these structural and functional benefits, supporting an NGF-dependent Akt/mTOR axis rather than non-specific effects [39].

*Nauclea pobeguinii* stem-bark extracts reduced hyperalgesia and pro-inflammatory cytokines while increasing sciatic-nerve NGF and IGF in STZ-diabetic mice [40]. Finally, a combination approach, mesenchymal stem cells plus resveratrol, outperformed either monotherapy in type-1 diabetic neuropathy, improving glycemic control and neuropathic endpoints, increasing sciatic-nerve NGF and myelin basic protein, and yielding larger axonal caliber and thicker myelin with reduced NF-κB immunoreactivity [41].

Consistently, a phenotyped cross-sectional study in Mexican adults with type 2 diabetes, including neuropathic subtypes, non-neuropathic diabetics and non-diabetic controls, showed markedly reduced circulating NGF in all diabetic groups versus controls, with no clear separation among neuropathy phenotypes; neuropathic cohorts also exhibited higher ICAM-1/VCAM-1/E-selectin and lower eGFR, linking NGF deficiency with systemic inflammation and endothelial dysfunction [42].

Clinicopathological data further associate reduced NGF with diminished TrkA and p75NTR and neuronal loss, in line with advanced trophic failure [43].

Two additional strands help reconcile why some studies report higher NGF while others show lower NGF. First is temporal and compartmental heterogeneity. The same animal can display early mechanical allodynia driven by NGF/TrkA upregulation in DRG/skin that later, as disease advances, gives way to trophic insufficiency in DRG, sciatic nerve, dorsal horn or bone marrow; NGF levels will therefore vary with when and where sampling occurs [24,25]. Second, matrix–neuron interactions modulate neurotrophin efficacy. Glycation of laminin and fibronectin in diabetic endoneurium reduces neurotrophin-stimulated neurite outgrowth, an effect partly prevented by aminoguanidine and only partly overcome by exogenous NGF; adult *db/db* sensory neurons retain intrinsic growth deficits even with NGF present, which helps explain diminishing returns of NGF augmentation in chronic disease [44,45]. Taking together, the literature supports a stage and compartment specific view of NGF in DPN. Studies reporting higher NGF most often capture early, localized nociceptive sensitization, whereas studies reporting lower NGF are more typical of progression, when neurotrophic support wanes and degeneration emerges. Differences in species and strain, diabetes duration and severity, anatomic compartment sampled and the interventional context account for much of the apparent discordance, and assay variability likely contributes as well. This framework reconciles why some strategies that dampen early NGF overdrive relieve pain while others that restore NGF-dependent trophic signaling support structure and function later, and it clarifies why systemic NGF replacement has not yet shown clinical benefit in human DPN despite compelling mechanistic rationale (Table 1).

### 3.2. Diabetic Encephalopathy

Diabetic encephalopathy (DE) denotes the constellation of structural, neurochemical and cognitive alterations that emerge in chronic hyperglycaemia. Clinically, patients display impaired learning, memory and psychomotor speed, while neuroimaging reveals cortical thinning, hippocampal atrophy and disrupted white-matter integrity within a multifactorial pathophysiology driven by insulin resistance, neuroinflammation, oxidative stress and vascular injury [46]. In this context, Soligo et al. demonstrated in STZ-induced diabetes does not deplete total brain NGF but skews its processing; mature NGF falls, whereas 34/50-kDa proNGF isoforms accumulate from week 4 onward, lowering the NGF/proNGF ratio and tracking the loss of TrkA- and ChAT-positive basal-forebrain cholinergic neurons, indicating defective maturation of NGF during the early progression of experimental diabetic encephalopathy [47]. Complementing these findings, Protto et al. demonstrated that three weeks of low-frequency electroacupuncture normalizes NGF homeostasis in the septo-hippocampal pathway of STZ-induced diabetic rats reducing the neurotoxic proNGF-B variant, restoring the mNGF—proNGF balance, rescuing TrkA expression, replenishing ChAT-positive neurons and cholinergic fibre density and altogether supporting reversal of early cholinergic dysfunction linked to NGF dysmetabolism [48] (Table 2).

### 3.3. Diabetic Retinopathy and Diabetic Keratopathy

Diabetic retinopathy (DR) is a leading cause of blindness and has traditionally been viewed primarily as a microvascular complication of diabetes [49]. However, accumulating evidence highlights that neuronal and glial dysfunction may precede and actively contribute to the vascular lesions observed in DR. Early manifestations of DR involve apoptosis of retinal ganglion cells (RGCs), reactive changes in Müller glial cells and an increase in inflammatory mediators. As the disease advances, cumulative microvascular damage fosters ischemic conditions, prompting pathological neovascularization [50].

The retina operates as an integrated neurovascular unit, wherein neurons, glia and capillary networks maintain functional interdependence. Disruption of this intricate relationship in DR suggests that neurotrophic factors, pivotal in supporting neuronal integrity, might also modulate vascular homeostasis [51]. Among these, NGF has emerged as a central mediator linking neurodegenerative processes to microvascular pathology in DR. Under physiological conditions, a balanced NGF/proNGF ratio is crucial for maintaining retinal neuronal health and vascular integrity [52]. In patients with proliferative DR, cross-sectional data show higher NGF concentrations in both serum and tear fluid compared with controls and non-proliferative disease, with positive correlations to HbA1c and diabetes duration; these relationships support the idea that systemic and tear NGF may rise alongside advanced retinopathy and chronic hyperglycemia [53]. Mechanistically, in a preclinical study, diabetes-induced oxidative stress, particularly through peroxynitrite formation, disrupts the proteolytic conversion of proNGF to NGF, causing an accumulation of proNGF and a concomitant reduction of mature NGF [54]. This shift from neuroprotective NGF-TrkA signaling towards harmful proNGF-p75NTR signaling correlates strongly with retinal neurodegeneration and microvascular dysfunction. Additionally, p75NTR signaling in Müller glial cells instigates an inflammatory response through activation of NF-κB and subsequent release of pro-inflammatory cytokines such as TNF-α and IL-1β, determining blood-retinal barrier breakdown [19]. Finally, proNGF has been shown to promote pathological retinal angiogenesis by activating TrkA and downstream MAPK signaling pathways, suggesting a mechanistic link between neurotrophin imbalance and proliferative changes in diabetic retinopathy. This inflammatory environment further exacerbates the reduction in NGF processing and accumulation of proNGF, creating a self-sustaining cycle of neuroinflammation and retinal damage [55]. Experimental studies strongly support this pathophysiological pathway, as administration of NGF in diabetic rat models demonstrated significant protection against neuroretinal apoptosis and vascular damage [56]. Similarly, enhancing NGF signaling through carbamazepine treatment in diabetic mice activated the PI3K/Akt/mTOR pathway, substantially reducing neuronal damage [57]. In vitro, NGF protected cultured retinal ganglion cells against palmitic acid-induced apoptosis via PI3K/Akt and ERK1/2 signaling [58] (Figure 2).

Clinical translation potential was demonstrated by topical NGF applications in diabetic mouse models, significantly improving both neuronal and vascular integrity through local neurotrophic signaling enhancement [59]. Targeting the detrimental proNGF-p75NTR pathway, Elshaer et al. showed that LM11A-31-mediated modulation of p75NTR prevented vascular leakage and inflammation in diabetic mice by suppressing RhoA kinase and NF-κB signaling [60]. Pharmacological inhibition of p75NTR using a selective antagonist in experimental models of diabetic retinopathy effectively prevented the pathological effects driven by proNGF. Blocking this signaling axis reduced vascular permeability, attenuated glial activation, limited neuronal apoptosis and suppressed the expression of inflammatory cytokines such as TNF-α and IL-1β. These findings confirm that proNGF, through p75NTR activation, orchestrates key paracrine mechanisms underlying the vascular, inflammatory and neurodegenerative components of the diabetic retina [61]. Genetic deletion of p75NTR in diabetic mice reduces retinal acellular capillary formation, highlighting the receptor’s critical role in mediating vascular damage through pro-apoptotic and inflammatory signaling pathways activated by proNGF [62]. In preclinical diabetic models, the intravitreal administration of mesenchymal stromal cells, either bone marrow-derived or from human umbilical sources, has shown to restore retinal homeostasis by creating a neuroprotective microenvironment rich in trophic factors, including NGF [63,64]. Additional studies by Saleh et al. and Ali et al. highlighted indirect protective effects via RAGE inhibition and mobilization of endothelial progenitor cells, respectively, both involving NGF-related angiogenic and inflammatory modulation [54,65]. This finding suggests that NGF could serve as a reliable, non-invasive biomarker for the early identification of retinal neurovascular dysfunction, as well as for tracking disease progression and evaluating therapeutic response in diabetic patients.

Diabetic neurotrophic keratopathy is a degenerative corneal condition caused by trigeminal nerve impairment, often secondary to DN, resulting in reduced corneal sensitivity, poor epithelial healing and risk of ulceration [66]. Under normal conditions, corneal epithelial, stromal and endothelial cells produce NGF and express its receptors TrkA and p75NTR and NGF–TrkA signaling is critical for corneal innervation and epithelial integrity [67]. When this axis is disrupted, epithelial homeostasis becomes compromised.

At the cellular level, NGF binding to TrkA activates pro-survival signaling cascades PI3K/Akt and ERK1/2, promoting corneal epithelial cell proliferation, migration and survival (Figure 2). Concomitantly, NGF triggers an anti-inflammatory response in surface inducing production of IL-1 receptor antagonist and IL-10 and dampens NF-κB activation, thereby reducing hyperglycemia-driven corneal inflammation [68]. These deficits can be rescued by blocking NGF activity accelerating re-epithelialization.

In STZ-induced diabetic mice, diabetes significantly altered the density and infiltration of DCs, a major source of ciliary neurotrophic factor (CNTF), by disrupting their neural communications. These changes resulted in reduced availability of DC-derived trophic support to corneal nerves, thereby contributing to diabetic corneal neuropathy [69].

Topical administration of recombinant human NGF (rhNGF) has shown encouraging results, enhancing corneal epithelial healing and restoring sub-basal nerve density in animal and human studies [70,71]. In more advanced approaches, a single intrastromal injection of gene therapy vectors designed to express NGF has also proven effective in the preclinical model, offering sustained improvement in corneal healing and nerve regeneration [72,73]. These strategies go beyond symptomatic management and aim to address the underlying neurotrophic deficit. By re-establishing the physiological support required for corneal homeostasis, NGF-based treatments could represent a meaningful advance in the management of diabetic neurotrophic keratopathy (Table 3).

### 3.4. Diabetic Cardiomyopathy

DM is frequently associated with cardiac dysfunction, diminished myocardial perfusion and an increased risk of heart failure [74]. Emerging evidence indicates that NGF exerts cardioprotective effects beyond its established neurotrophic functions. A preclinical study evaluated whether NGF gene transfer could mitigate the onset and progression of diabetic cardiomyopathy (DCM) in a murine model of STZ-induced diabetes, compared to non-diabetic controls. The results demonstrated that diabetic mice treated with NGF gene therapy were protected against progressive cardiac dysfunction and left ventricular dilatation. In addition, NGF-treated animals exhibited preservation of myocardial microvascular density, improved perfusion, reduced interstitial fibrosis and significantly attenuated apoptosis in both endothelial cells and cardiomyocytes [75] (Table 4). Complementary studies strengthen these observations. In isolated diabetic mouse hearts exposed to ischaemia–reperfusion (I/R), adenoviral NGF overexpression restored the down-regulated transient receptor potential vanilloid-1 (TRPV1) channel and the associated neuropeptides CGRP and SP, thereby limiting infarct size and improving contractile recovery; these benefits were abolished by the CGRP receptor antagonist CGRP8-37 [76]. Exogenous NGF administration in STZ-diabetic rats also normalized regional sympathetic heterogeneity, reduced ventricular effective refractory-period dispersion and lowered the incidence of ventricular arrhythmia, indicating that NGF directly stabilize cardiac electrophysiology under diabetic conditions [77]. Conversely, excessive NGF driven sympathetic sprouting can be maladaptive after myocardial infarction. In non-diabetic infarcted rats, the dipeptidyl-peptidase-4 (DPP-4) inhibitor sitagliptin blunted NGF expression by raising interstitial adenosine, lowering xanthine-oxidase substrates and suppressing superoxide generation, ultimately attenuating sympathetic hyperinnervation and arrhythmic vulnerability [78]. A follow-up study showed that the same molecule further reduces NGF-induced sympathetic remodeling via a cAMP/PKA/CREB-dependent up-regulation of the antioxidant enzyme haem-oxygenase-1 (HO-1), providing an additional layer of anti-arrhythmic protection. Taken together, these findings highlight a dual axis, whereby NGF-TRPV1 signaling preserves microvascular integrity and limits I/R injury in diabetic hearts, while therapies such as DPP-4 inhibition can modulate NGF-driven sympathetic remodeling through redox-sensitive HO-1 pathways, offering novel diagnostic and therapeutic avenues for DCM [79] (Figure 2).

### 3.5. Urogenital Complications

DM is associated with a range of urogenital complications that are often underrecognized despite their significant clinical impact [80]. Among these, diabetic bladder dysfunction (DBD) is a common condition affecting over 50% of patients with long-standing and poorly controlled diabetes. DBD encompasses both storage and avoiding disorders. It typically begins with symptoms of overactive bladder (OAB), such as urgency and incontinence and gradually progresses to a hyposensitive, underactive bladder characterized by impaired voiding, urinary retention and recurrent infections [81]. Structural and functional remodeling of the bladder, including wall thickening, often results from chronic oxidative stress and inflammation. As diabetes advances, DBD can evolve into diabetic cystopathy, defined by detrusor underactivity and sensory impairment. NGF, synthesized by urothelial and smooth muscle cells, plays a critical role in maintaining bladder innervation and modulating urothelial signaling. Studies in STZ-induced diabetic rats have shown decreased NGF levels in bladder tissue and dorsal root ganglia, accompanied by increased urinary NGF excretion. This paradox is thought to result from inflammation-induced apoptosis and the downregulation of NGF receptors, leading to impaired neurotrophic support and afferent nerve dysfunction [82] (Figure 2).

Therapeutic interventions, including insulin and human amniotic fluid stem cell (hAFSC) therapy, have been shown to restore NGF expression and improve levels of CGRP and SP, supporting detrusor function and neurodegeneration. NGF secreted by hAFSCs may exert neurotrophic and anti-apoptotic effects, contributing to tissue repair in the diabetic bladder [83] (Table 5). Emerging evidence implicates proNGF and p75NTR in diabetic voiding dysfunction. Under diabetic oxidative stress, impaired conversion of proNGF to mature NGF leads to accumulation of proNGF, which binds to p75NTR and activates pro-apoptotic and pro-inflammatory pathways, including TNF-α and RhoA [84]. In experimental diabetic models, inhibition of proNGF or blockade of p75NTR ameliorated bladder morphological and functional damage, validating this axis as a promising therapeutic target. Supplementation with grape seed proanthocyanidin extract has also been shown to normalize the proNGF/NGF balance in diabetic rats [85]. Diabetes additionally compromises the male reproductive system, affecting both humans and animal models. It impairs spermatogenesis, decreases sperm count and motility, promotes apoptosis, induces seminiferous tubule atrophy and reduces sperm volume and testosterone levels [86]. NGF, along with vascular endothelial growth factor (VEGF), regulates the proliferation and differentiation of spermatogenic and supporting cells, including spermatogonia, Leydig cells and Sertoli cells. NGF facilitates sperm motility, acrosomal reaction and testosterone synthesis. Decreased NGF levels correlate with germ cell atrophy and may serve as a biomarker of testicular dysfunction in diabetic patients [87].

### 3.6. Pancreatic β Cells

Insulin-secreting β-cells, although accounting for <2% of pancreatic mass, are indispensable for systemic glucose homoeostasis, releasing insulin in proportion to ambient glycaemia. Phenotypically, they exhibit several neuron-like attributes such as ligand-evoked exocytosis, membrane depolarization by glucose or acetylcholine and expression of neurotransmitter enzymes, neurofilaments and adhesion molecule including neural cell adhesion molecule (N-CAM) [88]. Polak et al. first highlighted this neuro–endocrine convergence; the immature RINm5F β-cell line, which co-expresses p75NGF and TrkA, extended neurite-like processes in response to NGF or laminin, whereas the more differentiated 83TC3 line responded solely to laminin. Anti-NGF antibodies abolished the NGF-induced outgrowth, underscoring a developmental program shared with neurons [89]. Subsequently, Rosenbaum et al. demonstrated that pancreatic cells not only respond to NGF but also synthesize and secrete the protein in a glucose- and K^+^-depolarization dependent manner via TrkA signaling [90]. New evidence shows that under diabetic conditions, β-cell NGF levels fall and maintaining NGF is protective. Tao et al. demonstrated in diabetic mouse β-cells and human pancreas samples that NGF expression is markedly reduced in islets from diabetic respect non-diabetic donors. NGF knockdown in β-cells lowers Sirt1 expression, reduces autophagy flux and decreases insulin secretion, whereas NGF overexpression rescues these defects. In that study, inhibition of *Cdk5* restored NGF and Sirt1 levels and protected β-cells. Together these findings suggest an NGF/Sirt1 axis where NGF upregulates Sirt1 to maintain autophagy and cell survival, conversely, hyperglycemia-driven *Cdk5* activation suppresses NGF, impairing autophagy [91]. Complementing these observations, Pierucci et al. demonstrated in primary human islets, purified human β-cells and β-cell lines that β-cells produce bioactive NGF and co-express TrkA and p75NTR; withdrawal or neutralization of endogenous NGF precipitated rapid, transcription- and translation-independent apoptosis characterized by caspase-3 activation, JNK engagement and bad dephosphorylation, whereas recombinant NGF rescued viability, thereby establishing an autocrine NGF–TrkA survival axis in human β-cells [92]. More recent studies have refined this paradigm. Houtz et al. localized NGF inside perivascular contractile pericytes encircling the islet, while TrkA receptors reside on β-cells. Hyperglycaemia rapidly augments vascular NGF release, activates β-cell TrkA and amplifies glucose-stimulated insulin secretion (GSIS). Mechanistically, NGF binding induces TrkA autophosphorylation (notably at Tyr 794) and recruitment of phospholipase C-γ1 (PLC-γ1). Activated PLC-γ1 hydrolyzes phosphatidylinositol-4,5-bisphosphate (PIP_2_), generating inositol-1,4,5-trisphosphate (IP_3_) and diacylglycerol; IP_3_ mediates Ca^2+^ release from endoplasmic reticulum stores, elevating cytosolic Ca^2+^ levels. The resulting rise in intracellular Ca^2+^, together with PLC-γ1–dependent *Rac1* activation that disassembles the cortical F-actin barrier, promotes the exocytosis of insulin granules. Targeted deletion of vascular NGF or β-cell TrkA impairs GSIS and provokes glucose intolerance, whereas exogenous NGF enhances GSIS in human islets without elevating basal insulin output [88] (Figure 2).

NGF also confers on cytoprotection. Larrieta et al. showed that, in rats exposed to acute STZ injury, pancreatic β-cells exhibit an early and disproportionate increase in NGF expression and secretion, despite a marked reduction in insulin production. This pattern supports the concept of NGF as an endogenous, early stress-response signal aimed at preserving β-cell survival when cytotoxic damage first occurs [93]. More recently, single-nucleus multi-omic profiling of islets from high-fat diet (HFD) mice has defined a trajectory from functionally competent “beta-hi” cells to dysfunctional “beta-low” states, characterized by loss of β-cell identity and induction of ER-stress pathways. Within this framework, ligand–receptor analysis identified NGF, produced by non-β islet cells, as a protective paracrine cue. Recombinant NGF reduced ER-stress/UPR gene expression in HFD islets and in thapsigargin-treated MIN6 cells in a TrkA-dependent manner and systemic NGF administration in Akita mice ameliorated ER-stress–driven β-cell dysfunction, increasing pancreatic/serum insulin and lowering fasting glycaemia. Taken together, these findings suggest that NGF is acutely upregulated as an early β-cell stress signal after injury, while targeted reinforcement of NGF–TrkA signaling under chronic metabolic or genetic stress can mitigate ER-stress-mediated β-cell failure [94]. In STZ-diabetic rats, oral cinnamon lowered hyperglycaemia and body weight while raising NGF and TrkA in both islets and exocrine pancreas, implying NGF-linked cytoprotection that may limit diabetes-related complications [95]. NGF acts as a double-edged mediator in pancreatic disease. In islet transplantation, transient NGF preconditioning raises VEGF expression and improves islet survival in vitro. Yet the same NGF-primed grafts fare poorly when infused into the portal vein, undergoing early apoptosis and showing sub-optimal engraftment. This adverse effect seems confined to the hepatic setting, where grafts implanted under the kidney capsule or in prevascularized chambers, where inflammatory pressure is lower, retain their viability [96]. In pancreatic ductal adenocarcinoma, a hyperglycemic tumor microenvironment enhances tumor cell proliferation, invasion and perineural invasion by upregulating NGF expression and strengthening the functional crosstalk between cancer cells and surrounding nerves. Neutralization of NGF attenuates hyperglycemia-induced tumor aggressiveness and nerve-directed invasion, supporting NGF as a key mediator linking diabetic metabolic conditions to exacerbated perineural invasion, rather than indicating a simple loss of NGF signaling [97] (Table 6).

### 3.7. Pharmacological Studies in Humans

A small number of clinical trials have explored NGF-based interventions in patients with diabetes and its complications (Table 7). These studies have tested both direct administration of NGF and indirect strategies to modulate the NGF pathway. Overall, the translation of NGF therapies to the clinical setting has yielded mixed results, underscoring significant challenges in efficacy and safety that need to be addressed.

The pivotal phase III trial by Apfel et al. randomized 1019 patients with diabetic polyneuropathy to subcutaneous recombinant human NGF (rhNGF, 0.1 µg/kg, *n* = 504) or placebo (*n* = 515), administered three times weekly for 48 weeks. The primary endpoint, change in the Neuropathy Impairment Score in the Lower Limbs (NIS-LL), showed no significant difference between the rhNGF and placebo groups (mean difference −0.2 points; 95% CI −1.8 to 1.4; *p* = 0.25). Among secondary outcomes, only modest improvements were noted on patient global symptom assessments (*p* = 0.03) and in isolated questionnaire items such as leg pain (*p* = 0.05). Safety quickly emerged as a major concern: injection-site hyperalgesia and pain were frequently reported in the rhNGF arm and the trial’s completion rate was lower with rhNGF (83%) compared to placebo (90%). These findings ultimately led to the discontinuation of systemic rhNGF development in diabetes [98].

A complementary line of investigation has targeted NGF inhibition to blunt nociceptor sensitization in DPN. In phase II, randomized, double-blind, placebo-controlled trial, patients with moderate to severe DPNP received subcutaneous Fulranumab (1, 3, or 10 mg every 4 weeks) or placebo. The study was terminated early following an FDA class-wide clinical hold on anti-NGF antibodies, with 77 of the planned 200 participants enrolled in the intent to treat population. Despite truncation, the primary endpoint, change in average daily pain at week 12, showed a dose-response (one-sided *p* = 0.014); pairwise comparison indicated a 1.2-point greater reduction on the 0–10 scale for Fulranumab 10 mg versus placebo (*p* = 0.040). Reported treatment-emergent adverse events included arthralgia (11%), peripheral edema (11%) and diarrhea (9%); no joint replacements or deaths occurred in this DPN cohort. Thus, NGF antagonism demonstrates an analgesic signal in DPNP, but interpretation is constrained by early termination and the broader class-specific safety concerns that prompted the clinical hold; fully powered, longer-term studies with musculoskeletal monitoring would be required to clarify benefit–risk [99].

Beyond neuropathy, NGF has been tested for male reproductive and sexual outcomes in diabetes. In a randomized open-label 2-arm study of 148 men with T2D, sensorimotor polyneuropathy and erectile dysfunction (IIEF-5 < 21), participants received standard of care with insulin, rosuvastatin 10 mg and α-lipoic acid 600 mg IV; the treatment arm additionally received intramuscular NGF 18 mg daily for the duration of hospitalization, mean 10 days, range 3–27. Compared with controls, NGF significantly increased total testosterone (+3.90 nmol/L, 95% CI 3.13–4.66 vs. +1.21 nmol/L, 95% CI 0.57–1.85), free testosterone (+3.79 pg/mL, 95% CI 3.05–4.54 vs. +1.27 pg/mL, 95% CI 0.85–1.70) and IIEF-5 score (+1.84, 95% CI 1.21–2.47 vs. +0.24, 95% CI −0.24 to 0.73), all *p* ≤ 0.001 for interaction/time-by-group. In supportive in-vitro experiments, NGF mitigated methylglyoxal-induced mitochondrial dysfunction in Leydig cells and up-regulated StAR and CYP11A1, consistent with enhanced steroidogenesis. Adverse events were not systematically reported; the open-label design, short exposure and co-interventions limit causal inference, but the findings suggest a potential endocrine/sexual-function signal worthy of confirmation in blinded, longer-duration trials [100].

Alternative approaches have aimed to indirectly modulate NGF levels.

A phase II randomized controlled trial tested tocotrienol-rich vitamin E (Tocovid), 200 mg twice daily for 12 months in 88 patients with type 2 diabetes and neuropathy. Compared with placebo, Tocovid improved peroneal nerve conduction velocity (+1.3 m/s vs. −0.4 m/s, *p* < 0.01) and increased circulating NGF levels (+19%, 95% CI 8–29%). No major adverse events were reported [101]. In a phase IIa randomized trial of rosuvastatin (20 mg/day for 12 weeks, *n* = 116) showed improvements in neuropathy symptom scores and reductions in oxidative stress markers (malondialdehyde −52%, *p* < 0.001), but β-NGF concentrations remained unchanged at approximately 1.5 pg/mL. Rosuvastatin was well tolerated, with an adverse event profile similar to placebo [102].

Targeted local delivery of NGF has shown more promise. In the pivotal randomized, double-masked, vehicle-controlled trial of topical ocular rhNGF (Cenegermin) for neurotrophic keratitis, 11 of the 156 enrolled patients had diabetes as the underlying etiology of corneal neurotrophy. Among these, the therapeutic response mirrored the overall study population. By week 8, 70–74% of Cenegermin-treated eyes achieved complete corneal healing compared to 43% in the vehicle group, with a median healing time of 2–3 weeks. Notably, over 96% of responders in the Cenegermin arm maintained epithelial integrity at 48 weeks. Treatment was well tolerated, with adverse events mostly limited to mild and transient ocular discomfort, and no serious events reported [11].

Local NGF therapy has also been explored in a small-scale setting. Generini et al. reported outcomes from three patients with chronic diabetic foot ulcers treated with topical NGF solution. All three refractory ulcers achieved complete closure within 8–12 weeks, accompanied by histological evidence of robust angiogenesis and nerve sprouting at the wound site. No adverse events occurred during topical NGF treatment. Although this uncontrolled case series is limited in scope, it suggests that localized NGF delivery may promote tissue repair while avoiding the systemic safety issues observed with circulating NGF exposure [103].

Taken together, pharmacological human studies of the NGF axis in diabetes remain heterogeneous and context dependent. Systemic rhNGF has not shown reproducible benefits in diabetic neuropathy and was hampered by tolerability issues. Anti-NGF therapy (Fulranumab) demonstrates a dose-responsive analgesic effect in painful DPN but is constrained by class-wide safety concerns and early trial termination. Short-term systemic NGF in men with T2D and ED signals improvements in testosterone and sexual function, yet evidence is open-label, short-duration and confounded by co-treatments. By contrast, local NGF delivery shows consistent, clinically meaningful tissue-repair effects with acceptable safety. Future clinical development should prioritize tissue-targeted or compartment-restricted delivery, employ sensitive and domain-appropriate endpoints, and incorporate rigorous safety surveillance for known risks, alongside standardized reporting of quantitative effect sizes to clarify the therapeutic potential of NGF-based interventions across diabetic complications.

## 4. Conclusions: Translational Challenges from Bench to Bedside for NGF-Targeted Therapies

In conclusion, dysregulation of the NGF axis, characterized by an excess of proNGF and a deficiency of mature NGF, emerges as a common pathogenic substrate across diverse diabetic complications. This imbalance is implicated in diabetic peripheral and autonomic neuropathies, retinopathy, cardiomyopathy, urogenital dysfunction and even pancreatic β-cell failure. Restoration of NGF–TrkA signaling in animal models consistently confers neuroprotective, vasculoprotective and cytoprotective effects, whereas unchecked proNGF–p75NTR activity amplifies inflammation, apoptosis and microvascular injury. These convergent findings establish NGF as a mechanistic keystone in diabetes-related tissue damage and also as a viable target for intervention, as well as a candidate biomarker for early detection of tissue distress and for monitoring therapeutic responses. However, translating NGF-targeted interventions from bench to bedside presents fundamental challenges in formulation, delivery and exposure control. NGF is a large and labile protein with poor tissue penetrance and a short systemic half-life, meaning that clinically useful exposure is highly dependent on the delivery strategy [104]. In practical terms, systemic administration of NGF has proven difficult to optimize, for instance, subcutaneous courses of rhNGF in diabetic polyneuropathy were technically feasible but ultimately failed to meet their primary efficacy endpoint and were consistently associated with injection-site hyperalgesia and pain. This narrow tolerability window likely prevented achievement of therapeutically effective tissue exposure, as evidenced by the discontinuation of further development after the phase III trial [98]. By contrast, topical ocular rhNGF (Cenegermin) improved corneal healing versus placebo in randomized, double-masked trials of neurotrophic keratopathy, validating a tissue-targeted approach with mainly local, manageable adverse effects [11]. Similarly, for central nervous system targets, intranasal NGF delivery demonstrates strong biological plausibility and early clinical feasibility, including successful pilot studies in pediatric cohorts, although its absolute delivery efficiency remains heavily dependent on formulation and device optimizations [15,105]

In parallel, AAV2-NGF gene delivery to the basal forebrain established stereotactic feasibility and acceptable safety in phase-1 studies but did not improve cognition or prespecified biomarkers in a controlled randomized trial, underscoring unresolved issues of dose control, vector tropism, surgical burden and verification of on-target bioactivity [14].

These examples illustrate that route of administration is a critical determinant for NGF-based therapies and each route comes with unique challenges and opportunities.

Safety considerations further complicate NGF-targeted therapy development. Exogenous NGF administration is dose limited by nociceptive adverse effects and by metabolic side effects such as weight loss [106]. Conversely, inhibiting NGF can also produce safety issues; NGF neutralization with monoclonal antibodies, such as Tanezumab for osteoarthritis, has been linked to arthralgia and incidents of rapidly progressive osteoarthritis, prompting the need for stringent exposure management and imaging-based surveillance in any program that perturbs this pathway [107]. Thus, any NGF-targeted therapy must carefully balance efficacy with a tight safety margin and regulatory agencies have imposed strict monitoring requirements when manipulating this pathway [108].

Equally important, the development of NGF and proNGF as reliable biomarkers is hampered by technical limitations in current assays. Mature NGF and proNGF can cross-react in commonly used immunoassays which biases quantification unless the two are analytically separated and results are confirmed with orthogonal methods [109]. Furthermore, reported NGF levels have been highly inconsistent across different diabetic complications and biological sample matrices, ranging widely in magnitude and even direction of change. This variability reinforces the need to predefine the specific context of use for NGF measurements, clearly delineating the clinical setting and sample type in which NGF or proNGF is to be measured before such biomarkers can be interpreted meaningfully in practice [43].

Looking ahead, high-impact progress in NGF-based therapy will require a concerted effort to overcome these translational barriers. First, rigorous, model-informed dose-finding studies and head-to-head comparisons of delivery routes are needed to determine how to achieve optimal NGF target engagement in humans. Second, future trials must integrate selective and standardized bioassays that can separately quantify NGF and proNGF in biological samples according to international validation criteria, such as ICH M10 guidelines for bioanalytical method validation [110]. Finally, combining these robust biomarker readouts with appropriate clinical endpoints in longitudinal cohorts and adaptive trial designs will be crucial to demonstrate true target engagement, clinically meaningful benefits and acceptable safety margins for NGF-targeted therapies. Together, these strategies will help ensure that promising mechanistic insights about NGF translate into tangible improvements in the prevention and treatment of diabetes complications.

## Figures and Tables

**Table 1:** Studies addressing NGF signaling, neuropathic pain, neuroprotection, and therapeutic modulation in diabetic peripheral neuropathy.

Ref.	Title	Model/Population	Study	NGF/proNGF Role/Biological Finding	Intervention/Administration	Dose	Outcomes/Conclusions	AE
[24]	Increased Nerve Growth Factor Signaling in Sensory Neurons of Early Diabetic Rats Is Corrected by Electroacupuncture	STZ-induced adult rats	Preclinical study	Early diabetes increased NGF and TrkA/p75NTR signaling (DRG, sciatic nerve) with TRPV1 upregulation; EA normalizes these changes	Low frequency EA (ST36 and SP6), 2 Hz	An amount of 2 Hz; 6 sessions (2×/week × 3 weeks); 30 min per session	EA reduced hyperalgesia and normalized NGF/TrkA/p75/TRPV1 and stress-kinase activity	Not reported
[25]	Nerve growth factor mediates mechanical allodynia in a mouse model of type 2 diabetes	C57BLKS *db/db* mice	Preclinical study	NGF overexpression in DRG and skin with increased TrkA phosphorylation; NGF–SP co-expression during peak allodynia	Neutralizing anti-NGF IgG intraperitoneal during peak allodynia	A total of 10 mg/kg i.p. at 6 and 7 weeks of age	Anti-NGF reversed allodynia and reduced SP+ neurons	Not reported
[26]	Carvedilol Exerts Neuroprotective Effect on Rat Model of Diabetic Neuropathy	STZ-induced male Sprague rats	Preclinical study	Diabetes increased DRG NGF and oxidative stress; carvedilol normalized NGF and redox markers	Oral carvedilol vs. α-lipoic acid comparator	Carvedilol 1 or 10 mg/kg/day × 45 days; α-lipoic acid 100 mg/kg/day	Improved behavioral neuropathic end-points; reduced DRG NGF and oxidative stress	Not reported
[27]	p38 mediates mechanical allodynia in a mouse model of type 2 diabetes	*db/db* mice	Preclinical study	Enhanced NGF–p75NTR–p38 signaling in DRG with upregulated COX-2, iNOS, TNF-α; anti-NGF reduced p38 activation	Systemic anti-NGF antibody; intrathecal SB203580	anti-NGF i.p.; SB203580 intrathecal	NGF/p38 pathway drives mechanical allodynia; blocking NGF or p38 reverses pain	Not reported
[28]	NGF/p38 signaling increases intraepidermal nerve fiber densities in painful neuropathy of type 2 diabetes	C57BLKS *db/db* mice vs. db/+ controls	Preclinical study	Transient increase of NGF-responsive peptidergic IENFs and NGF–p38-mediated sprouting during pain phase	Anti-NGF antibody i.p.; p38 inhibitor SB203580 intrathecal	Anti-NGF 10 mg/kg i.p. weekly × 2; SB203580 intrathecal infusion 1 mg/mL for 1 week	Blocking NGF or p38 prevented mechanical allodynia and aberrant sprouting	Not reported
[29]	The different dynamic changes of NGF in the dorsal horn and DRG leads to hyperalgesia and allodynia in diabetic neuropathic pain	STZ-induced diabetic rats	Preclinical study	NGF decreased in DRG early and later in dorsal horn parallel to hyperalgesia/allodynia	Exogenous mouse NGF i.p. started 2 weeks post-STZ	Either 4 or 20 μg/kg/day i.p. for 14 days	Exogenous NGF improved pain thresholds dose-dependently	Not reported
[30]	Does Endurance Training Compensate for Neurotrophin Deficiency Following Diabetic Neuropathy?	STZ-induced diabetic rats	Preclinical study	Diabetes reduced NGF/BDNF in roots; endurance training markedly increased NGF and partially restored BDNF	Endurance treadmill running	A total of 5 days/week for 6 weeks	Improved nerve conduction and thermal hyperalgesia	Not reported
[31]	Exercise May Increase Oxidative Stress in the Sciatic Nerve in Streptozotocin-Induced Diabetic Rats	STZ-induced diabetic rats	Preclinical study	DM increased sciatic-nerve NGF/BDNF; exercise improved MNCV but did not significantly change NGF/BDNF; oxidative stress marker 4-HNE increased with exercise	Treadmill exercise for 6 weeks vs. sedentary DM and controls	Treadmill training protocol per Methods	Exercise alleviated DM-induced slowing of MNCV, with mixed oxidative stress profile (↑ 4-HNE).	Not reported
[32]	Nerve growth factor gene therapy improves bone marrow sensory innervation and nociceptor-mediated stem cell release in a mouse model of type 1 diabetes with limb ischaemia	STZ-induced CD1 mice; in vitro PC12 assays	Preclinical study	T1D reduced BM innervation; Ad-hNGF restored sensory fibers and TrkA/Akt signaling	Single systemic injection of Ad-hNGF vs. β-gal control	A total of 1.5 × 10^9^ viral particles in 100 μl	Improved NK1R^+^ cell mobilization and limb blood-flow recovery	Not reported
[33]	The Correlation between Malondialdehyde and Nerve Growth Factor Serum Level with Diabetic Peripheral Neuropathy Score	Adults with diabetes (*n* = 30); DPN by MNSI criteria	Observational clinical study	Serum NGF inversely correlated with neuropathy severity; MDA positively correlated	N/A	N/A	Low NGF/high MDA associated with worse DPN; NGF showed stronger association	N/A
[34]	Heparin-Poloxamer Thermosensitive Hydrogel Loaded with bFGF and NGF Enhances Peripheral Nerve Regeneration in Diabetic Rats	STZ-induced diabetic rats	Preclinical study	Sustained local NGF + bFGF delivery activated MAPK/ERK, PI3K/Akt, JAK/STAT3 and enhanced regeneration	Perilesional injection of HP hydrogel loaded with NGF + bFGF (vs free GFs)	Single orthotopic HP injection	Improved functional recovery, axonal regeneration/myelination and muscle preservation vs. controls	Not reported
[35]	Transplantation of human dental pulp stem cells ameliorates diabetic polyneuropathy in streptozotocin-induced diabetic nude mice: the role of angiogenic and neurotrophic factors	STZ-induced diabetic nude mice	Preclinical study	hDPSCs localized in injected muscle and secreted human NGF and VEGF; NGF/VEGF neutralization abrogated therapeutic benefit	A total of 1 × 10^5^ hDPSCs injected into unilateral hind-limb skeletal muscle at 10 sites; contralateral limb received saline	hDPSCs: 1 × 10^5^ cells/limb in 0.2 mL; Neutralizing Ab (NGF or VEGF) 0.5 μg/mouse/day for 4 weeks	Improved MNCV/SNCV and sciatic blood flow; increased capillary/muscle bundle ratio; effects suppressed by NGF/VEGF antibodies	Not reported
[36]	Therapeutic efficacy of bone marrow-derived mononuclear cells in diabetic polyneuropathy is impaired with aging or diabetes	STZ-diabetic rats; BM-MNCs from young non-diabetic vs. adult non-diabetic vs. adult diabetic rats	Preclinical study	BM-MNCs from adult diabetic rats expressed less NGF and bFGF and had fewer CD29^+^/CD90^+^ MSC-like cells; therapeutic efficacy diminished	Intramuscular injection of BM-MNCs into hind-limb skeletal muscles	A total of 0.5 mL, 1 × 10^8^ cells	Young donor BM-MNCs improved thermal sensation, sciatic blood flow, and NCV; adult/diabetic donor BM-MNCs showed no benefit	Not reported
[37]	Effect of Metformin on Schwann Cells under Hypoxia	Primary Schwann cells	Preclinical study	Hypoxia decreased NGF; metformin increased NGF/BDNF/GDNF expression and secretion via AMPK	Metformin during hypoxia/reperfusion	An amount of 2 mM metformin in culture	Reduced apoptosis and preserved SC migration/viability; effects partly AMPK-dependent	N/A
[38]	Neuroprotective effects of *Gymnema sylvestre* on streptozotocin-induced diabetic neuropathy in rats	STZ-induced Wistar rats	Preclinical study	Diabetes lowered NGF; Gymnema increased NGF/IGF-1 and antioxidant enzymes	Oral *G. sylvestre* leaf extract	Either 50 or 100 mg/kg/day for 5 weeks	Improved conduction velocity, pain thresholds, and sciatic nerve histology; ↑ NGF	Not reported
[39]	Improvement of diabetes-induced spinal cord axon injury with taurine via NGF-dependent Akt/mTOR pathway	STZ-induced diabetic rats; primary cortical neurons and VSC4.1 cells	Preclinical study	Taurine upregulated NGF and GAP-43 and activated Akt/mTOR, promoting neuritogenesis and attenuating axonal damage	Oral taurine in drinking water starting 3 days post-STZ	Either 0.5%, 1.0%, or 2.0% taurine ad libitum for 8 weeks	Dose-responsive improvements in morphology and neurological function via NGF-dependent Akt/mTOR signaling	Not reported
[40]	Antihypernociceptive and Neuroprotective Effects of the Aqueous and Methanol Stem-Bark Extracts of *Nauclea pobeguinii* on STZ-Induced Diabetic Neuropathic Pain	STZ-induced diabetic mice	Preclinical study	Diabetes dysregulated NGF and other neurotrophic factors; extracts modulated NGF and reduced nociception	Stem-bark extracts	Multiple doses	Reduced hypernociception and enhanced neuroprotection	Not reported
[41]	The combined effect of mesenchymal stem cells and resveratrol on type 1 diabetic neuropathy	STZ-induced diabetic rats	Preclinical study	MSC + resveratrol increased NGF in peripheral nerves and reduced oxidative stress	MSCs plus oral resveratrol	Resveratrol 200 mg/kg/day × 56 days; MSC dosing per Methods	Higher NCV and improved myelinated fiber density vs. monotherapies	Not reported
[42]	Neuropathy-specific alterations in a Mexican population of diabetic patients	A total of 243 T2D patients (217 with neuropathy) + 26 non-diabetic controls + 375 non-diabetic	Observational clinical study	Plasma NGF reduced in diabetes and further in neuropathy; increased ICAM/VCAM/E-selectin; lower GFR, especially motor neuropathy	N/A	N/A	Circulating NGF reduction associates with neuropathy phenotype and endothelial activation	N/A
[43]	Diagnostic Significance of Serum Levels of Nerve Growth Factor and Brain Derived Neurotrophic Factor in Diabetic Peripheral Neuropathy	An amount of 65 DPN, 83 T2DM without DPN, 110 healthy controls	Diagnostic observational study	Serum NGF and BDNF lowest in DPN; correlated with HbA1c, C-peptide and microalbuminuria	N/A	N/A	ROC: NGF AUC 0.933 (cut-off: 50.25 pg/mL); BDNF AUC 0.925; combined markers improved accuracy	N/A
[44]	Advanced glycation end products in extracellular matrix proteins contribute to the failure of sensory nerve regeneration in diabetes	STZ-diabetic rats + DRG neuron cultures	Preclinical study	AGE-modified ECM impairs neurite outgrowth; NGF supplementation restores neuritogenesis despite glycated ECM	Exogenous NGF added to DRG cultures plated on glycated ECM	NGF 10 ng/mL (in vitro)	NGF restored neurite branching on glycated fibronectin; aminoguanidine prevented glycation-induced inhibition	Not reported
[45]	Sensory neuron cultures derived from adult *db/db* mice as a simplified model to study type-2 diabetes-associated axonal regeneration defects	DRG neurons from adult *db/db* vs. db/+ mice	Preclinical study	Reduced neurite outgrowth and blunted response to NGF/laminin in *db/db* neurons	NGF supplementation in culture	An amount of 5 µg/mL NGF	NGF increased outgrowth but less than controls; model suitable for testing regenerative therapies	N/A

Abbreviations: AE, adverse events; AGE, Advanced Glycation End products; Ad-hNGF, Adenoviral Human Nerve Growth Factor construct; AGE, Advanced Glycation End product; BDNF, Brain-Derived Neurotrophic Factor; bFGF, basic Fibroblast Growth Factor; BM, Bone Marrow; DM, Diabetes Mellitus; BM-MNCs, bone marrow-derived mononuclear cells; DPN, Diabetic Peripheral Neuropathy; DRG, Dorsal Root Ganglion; EA, Electroacupuncture; ECM, Extracellular Matrix; GDNF, Glial cell line-Derived Neurotrophic Factor; GFs, growth factors; HbA1c, glycated hemoglobin; hDPSC, human Dental Pulp Stem Cell; HP hydrogel, Heparin–Poloxamer thermosensitive hydrogel; ICAM, Intercellular Adhesion Molecule; i.p., intraperitoneal; IENFs, intraepidermal nerve fibers; MDA, Malondialdehyde; MNCs, Mononuclear Cells; MNCV/SNCV, Motor/Sensory Nerve Conduction Velocity; MNSI, Michigan Neuropathy Screening Instrument; MSC, Mesenchymal Stem Cell; N/A, not available; NCV, Nerve Conduction Velocity; SB203580, selective p38 MAPK inhibitor; SP, Substance P; STZ, Streptozotocin; T1D, Type 1 Diabetes; T2DM, Type 2 Diabetes Mellitus; VCAM, Vascular Cell Adhesion Molecule. Symbols: ↑, increased levels/expression.

**Table 2:** Studies linking altered NGF/proNGF balance to neurodegeneration, cholinergic dysfunction and cognitive impairment in diabetic encephalopathy.

Ref.	Title	Model/Population	Study	NGF/proNGF Role/Biological Finding	Intervention/ Administration	Dose	Outcomes/Conclusions	AE
[47]	The mature/proNGF ratio is decreased in the brain of diabetic rats: analysis by ELISA methods	STZ-induced diabetic rats	Preclinical study	In diabetic brain regions, mNGF decreased while proNGF increased, lowering the mNGF/proNGF ratio and reducing ChAT^+^ and p75NTR^+^ neurons in cortex and hippocampus	N/A (observational)	N/A	From 4 to 8 weeks, decreased mNGF and increased proNGF impaired NGF maturation, leading to cholinergic dysfunction and early diabetic encephalopathy	N/A
[48]	Electroacupuncture in rats normalizes the diabetes-induced alterations in the septo-hippocampal cholinergic system	STZ-induced diabetic rats	Preclinical study	Diabetes disrupted NGF metabolism in the basal forebrain–hippocampal circuit, with elevated proNGF, reduced mature NGF and TrkA/ChAT signaling, leading to impaired synaptic plasticity, learning, and memory	Low frequency EA at acupoints	An amount of 2 Hz EA, 20 min/session, 2×/week for 3 weeks, starting in week 3 of diabetes	In untreated diabetic rats, memory loss was linked to proNGF accumulation, reduced TrkA and impaired cholinergic signaling, while electroacupuncture restored NGF balance, TrkA/ChAT expression, cholinergic phenotype, and hippocampal plasticity, improving memory	No adverse effects observed

Abbreviations: AE, adverse events; ChAT, Choline Acetyltransferase; EA, Electroacupuncture; mNGF, mature Nerve Growth Factor; N/A, not available; STZ, Streptozotocin.

**Table 3:** Studies on NGF-related mechanisms and therapeutic strategies in diabetic retinopathy and keratopathy.

Ref.	Title	Model/ Population	Study	NGF/proNGF Role/Biological Finding	Intervention/Administration	Dose	Outcomes/Conclusions	AE
[53]	Serum and tear levels of nerve growth factor in diabetic retinopathy patients	A total of 254 patients with DR (103 NPDR, 151 PDR) vs. 71 non-diabetic controls	Cross-sectional observational clinical study	Serum and tear NGF increased with DR severity (highest in PDR) and correlated with diabetes duration, HbA1c, blood glucose and nephropathy; strong tear–serum concordance	N/A	N/A	Supports NGF as a biomarker of DR severity	N/A
[54]	Diabetes-induced peroxynitrite impairs the balance of pro-nerve growth factor and nerve growth factor, and causes neurovascular injury	STZ-induced diabetic rats; Vitreous from patients with PDR; Cultured retinal cells	Preclinical study	Peroxynitrite nitrates MMP-7, impairing NGF maturation and causing proNGF accumulation → p75NTR activation, neurodegeneration and vascular injury	Atorvastatin; FeTPPS peroxynitrite scavenger	Atorvastatin 10 mg/kg/day p.o.; FeTPPS 15 mg/kg/day i.p.	Targeting peroxynitrite restored NGF, reduced proNGF, and decreased BRB leakage and neuronal apoptosis	No adverse effects reported
[19]	Modulation of p75NTR prevents diabetes- and proNGF-induced retinal inflammation and BRB breakdown in mice and rats	STZ-diabetic wild-type and p75NTR−/− mice; rat intravitreal proNGF model	Preclinical study	Diabetes reduced mature NGF and increased proNGF, activating p75NTR and driving inflammation and BRB leakage; p75NTR blockade preserved retinal integrity	Intravitreal proNGF (viral vector) ± p75NTR inhibitor peptide; genetic deletion (p75NTR−/−)	pGFP–proNGF + LV-scrambled p75NTR (5000 IFU/eye) and pGFP–proNGF + LV-shRNA p75NTR (5000 IFU/eye)	p75NTR modulation restored NGF/proNGF balance, reduced neuroinflammation, and protected BRB and ganglion cells	No adverse effects reported in animals
[56]	Nerve growth factor prevents both neuroretinal programmed cell death and capillary pathology in experimental diabetes	STZ-induced diabetic rats	Preclinical study	Diabetes increased RGC apoptosis and capillary pathology; exogenous NGF prevented neuronal death and vascular lesions	Systemic NGF administration	5 mg/kg three times per week	NGF provided neuroprotection (↓ RGC apoptosis) and vasculoprotection (↓ acellular capillaries)	No adverse effects reported
[57]	Carbamazepine Alleviates Retinal and Optic Nerve Neural Degeneration in Diabetic Mice via Nerve Growth Factor-Induced PI3K/Akt/mTOR Activation	Alloxan-induced diabetic mice	Preclinical study	Diabetes suppressed NGF/TrkA–PI3K/Akt/mTOR and increased caspase-3; carbamazepine restored NGF signaling and reduced apoptosis	Oral CBZ	Either 25 or 50 mg/kg/day × 4 weeks	Reduced retinal vacuolisation and optic-nerve atrophy; improved structure and function via NGF-pathway activation	No adverse effects reported
[58]	NGF protects against palmitic-acid injury in retinal ganglion cells	RGC-5 cells	Preclinical study	Palmitate increased ROS and apoptosis; NGF activated PI3K/Akt and ERK to reduce both; pathway inhibitors abolished protection	NGF ± PI3K/Akt or ERK inhibitors in culture	NGF 50 ng/mL	NGF enhanced cell viability and reduced oxidative stress and apoptosis via PI3K/Akt and ERK signaling	N/A
[59]	Topical nerve growth factor prevents neurodegenerative and vascular stages of diabetic retinopathy	Ins2Akita mice, wild type (C57BL/6J) mice	Preclinical study	Topical rhNGF preserved RGCs, reduced apoptosis and glial activation, and limited vascular acellularity	Topical rhNGF eye drops	An amount of 180 mg/mL	rhNGF improved retinal morphology and function and prevented neurovascular injury with long-term protection	No adverse effects reported
[60]	Modulation of the p75 neurotrophin receptor using LM11A-31 prevents diabetes-induced retinal vascular permeability in mice via inhibition of inflammation and the RhoA kinase pathway	STZ-induced diabetic C57BL/6J mice	Preclinical study	In diabetes, proNGF accumulation with reduced NGF and increased p75NTR activated RhoA, driving vascular leakage and inflammation	LM11A-31 (p75NTR modulator/proNGF antagonist)	An amount of 50 mg/kg/day for 4 weeks	LM11A-31 reduced proNGF, restored NGF balance, preserved BRB integrity, and suppressed VEGF and inflammatory signaling	No adverse effects reported
[61]	p75NTR and Its Ligand ProNGF Activate Paracrine Mechanisms Etiological to the Vascular, Inflammatory, and Neurodegenerative Pathologies of Diabetic Retinopathy	STZ-diabetic mice; human DR vitreous; retinal endothelial & glial cultures	Preclinical study	ProNGF accumulation with reduced mature NGF activated p75NTR, triggering inflammation, vascular leakage, and neuronal apoptosis.	Anti-proNGF antibody; p75NTR inhibitor (THX-B)	An amount of 2 µg THX-B or 2 µg anti-proNGF NGF30 mAb	Blocking proNGF/p75NTR preserved BRB and neuronal survival by reducing leakage, inflammation, and apoptosis	No adverse effects reported
[62]	Deletion of the Neurotrophin Receptor p75NTR Prevents Diabetes-Induced Retinal Acellular Capillaries in Streptozotocin-Induced Mouse Diabetic Model	STZ-induced diabetic mice	Preclinical study	Diabetes-induced proNGF/NGF imbalance activated p75NTR and drove vascular damage; deletion protected against apoptosis, inflammation, and pericyte/endothelial loss	Genetic deletion (p75NTR)	N/A	p75NTR deletion prevented acellular capillaries and preserved retinal vasculature	N/A
[63]	Intravitreal administration of multipotent mesenchymal stromal cells triggers a cytoprotective microenvironment in the retina of diabetic mice	STZ-induced diabetic mice	Preclinical study	MSC paracrine secretome (including NGF) supported retinal neuroprotection	Single intravitreal injection of MSCs	A total of 2 × 10^5^ cells/eye	Reduced retinal apoptosis and RGC loss; preserved retinal function via trophic mechanisms	No adverse effects reported
[64]	A comparative study on the transplantation of different concentrations of human umbilical mesenchymal cells into diabetic rats	STZ-induced diabetic rats	Preclinical study	Higher hUC-MSC dose increased retinal NGF and histological protection	Intravitreal transplantation of hUC-MSCs	A total of 4 × 10^5^ cells/eye and 8 × 10^5^ cells/eye	Dose-dependent retinal protection with increased NGF expression	No adverse effects reported
[65]	Inhibition of Receptor for Advanced Glycation End Products as New Promising Strategy Treatment in Diabetic Retinopathy	Alloxan-induced diabetic Wistar rats	Preclinical study	AGE–RAGE activation increased oxidative stress, VEGF and GFAP and reduced NGF; RAGE inhibition reversed these effects and limited pericyte damage and neovascularisation	Anti-RAGE antibody	A total of 1 μg/μL, 10 μg/μL or 100 μg/μL	RAGE inhibition lowered glucose/HbA1c, restored retinal NGF, reduced GFAP/VEGF/RAGE and suppressed disease progression	N/A
[70]	Effect of recombinant human nerve growth factor treatment on corneal nerve regeneration in patients with neurotrophic keratopathy	Adults with stage 2–3 neurotrophic keratopathy (including nine diabetic patients)	Clinical study	Topical rhNGF promoted corneal reinnervation and enhanced epithelial healing	Cenegermin eye drops	Cenegermin 0.002%	Increased corneal nerve fibre density and sensitivity; accelerated epithelial closure; long-term improvement	Mild ocular pain, transient hyperemia
[71]	Neurotrophic Keratopathy in Systemic Diseases: A Case Series on Patients Treated With rh-NGF	Clinical Case series	Observational clinical study	Topical rhNGF enhanced epithelial healing and improved corneal sensitivity	Cenegermin ophthalmic solution	A total of 20 μg/mL; 1 drop 6×/day for 8 weeks	Majority achieved re-epithelialisation and functional improvement	Mild, transient eye discomfort; generally well tolerated
[72]	Preventing and treating neurotrophic keratopathy by a single intrastromal injection of AAV-mediated gene therapy	*db/db* mice	Preclinical study	AAV-mediated NGF delivery sustained NGF expression and promoted corneal nerve regeneration and reinnervation	Single intrastromal injection of AAV-NGF	A total of 1 × 10^9^ vector genomes AAV	Single administration produced long-term nerve regrowth, reduced epithelial defects and durable efficacy	No adverse effects reported
[73]	Long-term Nerve Regeneration in Diabetic Keratopathy Mediated by a Novel NGF Delivery System	STZ-induced diabetic rats	Preclinical study	Diabetes reduced corneal NGF and nerve density; sustained NGF delivery restored trophic support and promoted long-term corneal innervation and epithelial repair	Novel biodegradable NGF delivery matrix (hydrogel/implant) applied to cornea or stroma	Controlled-release NGF microdose maintained locally for several weeks	Sustained NGF delivery enhanced corneal reinnervation, epithelial healing, and sensory recovery, maintaining long-term regeneration without adverse effects	No adverse reactions

Abbreviations: AAV, Adeno-Associated Virus; AE, adverse events; AGE, Advanced Glycation End-products; BRB, Blood–Retinal Barrier; CBZ, Carbamazepine; DR, Diabetic Retinopathy;GFAP, Glial Fibrillary Acidic Protein; FeTPPS, 5,10,15,20-Tetrakis(4-sulfonatophenyl)porphyrinato iron(III) chloride; HbA1c, glycated hemoglobin; hUC-MSC, Human umbilical cord mesenchymal stem cells; i.p., intraperitoneal; mAb, monoclonal Antibody; MMP-7, Matrix Metallopeptidase-7; MSC, Mesenchymal Stem Cell; N/A, not available; NGF, Nerve Growth Factor; NPDR, Non-Proliferative Diabetic Retinopathy; p75NTR, neurotrophin receptor p75; PDR, Proliferative Diabetic Retinopathy; p.o., per os; RAGE, Receptor for Advanced Glycation End-products; RGC-5, Retinal Ganglion Cells-5; rhNGF, recombinant human Nerve Growth Factor; ROS, Reactive Oxygen Species; STZ, Streptozotocin. Symbols: ↓, decreased levels/expression.

**Table 4:** Experimental and clinical studies on NGF modulation in diabetic cardiomyopathy and its role in cardiac function, remodeling and arrhythmia susceptibility.

Ref.	Title	Model/ Population	Study	NGF/proNGF Role/Biological Finding	Intervention/ Administration	Dose	Outcomes/Conclusions	AE
[75]	NGF gene therapy using adeno-associated viral vectors prevents cardiomyopathy in type 1 diabetic mice	STZ-induced diabetic adult rats	Preclinical study	Cardiac NGF depletion in diabetes provokes LV dysfunction, fibrosis, apoptosis and microvascular rarefaction; restoration of myocardial NGF reverses these processes	AAV-hNGF gene transfer vs. AAV-β-gal control, administered 2 weeks after diabetes induction	A total of 1.5 × 10^12^ viral particles in 100 μL	*NGF* gene transfer preserved LV structure and function and prevented dilation, fibrosis, apoptosis and capillary loss, halting diabetic cardiomyopathy progression	No adverse effects reported
[76]	Nerve growth factor rescues diabetic mice heart after ischemia/reperfusion via up-regulation of the TRPV1 receptor	STZ-induced diabetic adult mice	Preclinical study	Diabetes reduces cardiac NGF, TRPV1, and neuropeptides (CGRP, SP); NGF gene transfer up-regulates TRPV1 and CGRP, improving post-I/R recovery. CGRP blockade abrogates benefit; capsaicin mimics TRPV1-dependent protection	Catheter-based LV injection of Ad-NGF; ex vivo perfusion ± CGRP antagonist, SP antagonist, or capsaicin	Ad-NGF 2.0 × 10^11^ pfu/mL, 35 µL into LV root; perfusate: CGRP8-37 10^−6^ M; RP67580 10^−6^ M; capsaicin 10^−6^ M	*NGF* gene therapy restored TRPV1/CGRP signaling and significantly improved LV function and reduced LDH release after I/R in diabetic hearts	No adverse effects reported
[77]	Exogenous NGF promotes the repair of cardiac sympathetic heterogeneity and electrophysiological instability in diabetic rats	STZ-induced Wistar rats	Preclinical study	Diabetes decreased LV NGF and TH with proximal-distal sympathetic heterogeneity and prolonged QT/QTd; targeted NGF replenishment restored regional NGF/TH and reduced ventricular arrhythmias	LSG injections of mouse NGF vs. saline control (4 injections over 4 weeks).	A total of 20 μg/kg NGF injected into LSG, once every 3 days × 4 injections (total 4 weeks).	NGF normalized sympathetic innervation and QT indices, reducing inducible ventricular arrhythmias and preventing CAN-related electrophysiological instability	No adverse effects reported
[78]	Sitagliptin attenuates sympathetic innervation via modulating ROS and interstitial adenosine in infarcted rat hearts	Male Wistar rats with MI by left coronary ligation	Preclinical study	Post-MI oxidative stress increases NGF and sympathetic sprouting; sitagliptin reduces ROS, down-regulates NGF, and limits hyperinnervation, lowering arrhythmia risk	Sitagliptin by oral gavage daily for 4 weeks post-MI; ex vivo confirmation with EHNA (adenosine deaminase inhibitor), DPCPX (A1 antagonist) and hypoxanthine	Sitagliptin 10 mg/kg/day (oral, 4 weeks). EHNA 5 μM, DPCPX 200 nM	Sitagliptin prevented arrhythmias by reducing ROS and myocardial NGF, limiting sympathetic sprouting and improving electrophysiological stability	No significant adverse effects reported
[79]	Dipeptidyl Peptidase-4 inhibition attenuates arrhythmias via a PKA-dependent pathway in infarcted hearts	Male Wistar rats with MI by left coronary ligation	Preclinical study	Sitagliptin decreased NGF and sympathetic hyperinnervation and reduced arrhythmic scores through a cAMP/PKA/CREB-dependent increase in HO-1 and antioxidant signaling	In vivo sitagliptin by gastric gavage 24 h post-MI for 4 weeks; ex vivo perfusion with PKA inhibitor (H-89), Epac inhibitor (brefeldin A), PKA agonist (N6-Bz-cAMP), Epac agonist (8-CPT-cAMP), CREB inhibitor (KG-501)	In vivo: sitagliptin 5 mg/kg/day × 4 weeks. Ex vivo: H-89 0.1 µM; brefeldin A 100 µM; N6-Bz-cAMP 1 mM; 8-CPT-cAMP 1 mM; KG-501 10 µM	Sitagliptin attenuated NGF-driven sympathetic reinnervation and ventricular arrhythmias via PKA/CREB-mediated HO-1 up-regulation and antioxidant effects	No significant adverse effects

Abbreviations: AAV-β-gal, Adeno-Associated Virus serotype-β-galactosidase; AAV-hNGF, Adeno-Associated Virus serotype–human nerve growth factor; AE, adverse events; CAN, cardiac autonomic neuropathy; DPCPX, 8-Cyclopentyl-1,3-dipropylxanthine; EHNA, Erythro-9-(2-hydroxy-3-nonyl)adenine; Epac, Exchange Protein directly Activated by cAMP; LV, Left ventricular; LSG, Left Stellate Ganglion; MI, Myocardial Infarction; NGF, Nerve Growth factor; STZ, Streptozotocin; TH, tyrosine hydroxylase.

**Table 5:** Studies on NGF alterations and therapeutic interventions in diabetic urogenital complications, including bladder dysfunction and testicular damage.

Ref.	Title	Model/ Population	Study	NGF/proNGF Role/ Biological Finding	Intervention/ Administration	Dose	Outcomes/ Conclusions	AE
[82]	Functional and Molecular Characterization of Hyposensitive Underactive Bladder Tissue and Urine in Streptozotocin-Induced Diabetic Rat	STZ-induced diabetic rats	Preclinical study	In vivo experimental study with cystometry, histology, Western blot, ELISA	Late-stage diabetic hyposensitive underactive bladder associated with decreased bladder NGF and markedly increased urinary NGF, alongside EP1/EP3 upregulation, inflammation and apoptosis; NGF as a potential non-invasive biomarker of advanced diabetic cystopathy	Intravesical PGE_2_ during CMG to test bladder responsiveness, 100 µM intravesical infusion	Confirms progressive hyposensitive underactive bladder in DM; identifies discordant tissue vs. urinary NGF (low bladder, high urine) and EP3 upregulation as key features; proposes urinary NGF as candidate biomarker and EP3 signaling as potential therapeutic target	N/A
[83]	Human amniotic fluid stem cell therapy can help regain bladder function in T2D rats	STZ-induced diabetic rats	Preclinical study	Diabetic rats exhibit bladder dysfunction with reduced bladder NGF, CGRP, SP and β-cell markers. Both insulin and hAFSCs treatment tend to restore NGF and sensory neuropeptide expression toward control levels, supporting partial recovery of neurotrophic support in diabetic bladder.	hAFSC intravenous injection (± insulin co-treatment)	hAFSCs: 1 × 10^6^ cells	Insulin therapy clearly improves cystometric parameters and bladder weight; hAFSCs show beneficial trends in bladder function and neurotrophic/inflammatory markers, suggesting a potential supportive role via NGF-related and paracrine mechanisms in type 2 diabetic cystopathy.	No adverse effect reported
[84]	Antagonism of proNGF or its receptor p75NTR reverses remodeling and improves bladder function in a mouse model of diabetic voiding dysfunction	STZ-induced diabetic mice	Preclinical study	Diabetes increased the proNGF/NGF ratio; blocking p75NTR or neutralising proNGF normalised the ratio and inflammatory signaling	Anti-proNGF monoclonal antibody or p75NTR antagonist (THX-B)	A total of 50 μg/mouse	Decreased bladder weight; improved compliance and contractility; reduced TNF-α levels	No adverse effect reported
[85]	Grape Seed Proanthocyanidin Extract Ameliorates Diabetic Bladder Dysfunction via the Activation of the Nrf2 Pathway	STZ-induced diabetic rats	Preclinical study	Diabetes decreased NGF and increased proNGF; GSPE reversed these changes in association with *Nrf2* pathway activation	Oral GSPE	A total of 250 mg/kg/day × 8 weeks	Improved bladder capacity and contractility, reduced oxidative stress, and restoration of the proNGF/NGF balance	No adverse effect reported
[87]	Potential Novel Biomarkers for Diabetic Testicular Damage in Streptozotocin-Induced Diabetic Rats: Nerve Growth Factor Beta and Vascular Endothelial Growth Factor	STZ-induced diabetic rats	Preclinical study	Reduced testicular NGFβ associated with structural/functional damage; VEGF changes paralleled tissue injury	N/A	N/A	NGFβ and VEGF emerged as potential biomarkers of diabetic testicular damage	N/A

Abbreviations: CGRP, Calcitonin Gene-Related Peptide; GSPE, Grape Seed Proanthocyanidin Extract; hAFSC, human Amniotic Fluid Stem Cells; N/A, not available; NGF, Nerve Growth Factor; p75NTR, neurotrophin receptor p75; STZ, Streptozotocin; SP, Substance P.

**Table 6:** Studies investigating NGF signaling in pancreatic β-cell biology, insulin secretion and β-cell adaptation in diabetes.

Ref.	Title	Model/Population	Study	NGF/proNGF Role/Biological Finding	Intervention/ Administration	Dose	Outcomes/Conclusions	AE
[89]	Nerve growth factor induces neuron-like differentiation of an insulin-secreting pancreatic beta cell line	Rat insulinoma β-cell lines RINm5F and βTC3; PC12 and hepatoma cells as controls	Preclinical study	RINm5F cells express p75 and Trk NGF receptors and respond to NGF with neurite-like outgrowth, neurofilament-positive processes, and induction of NGF-1A, indicating a functional NGF signaling axis and a neuroendocrine-like differentiation program in immature β-cells; βTC3 cells lack p75 and do not respond, suggesting NGF responsiveness is stage-dependent	NGF; anti-NGF antibody; laminin	NGF 50–100 ng/mL; anti-NGF Ab 500 ng/mL; laminin 10 µg/mL	NGF specifically induces neuron-like differentiation of immature β-cells via NGF receptors (blocked by anti-NGF); laminin induces processes in both lines. Findings support overlap between neuronal and β-cell developmental programs and suggest a potential role for NGF in pancreatic β-cell differentiation/regeneration	N/A
[90]	Pancreatic β-cells synthesize and secrete nerve growth factor	Rats islets/β-cells	Preclinical study	β-cells synthesize and secrete NGF in response to glucose and depolarization, with TrkA expression supporting an autocrine/paracrine regulatory loop	In vitro secretory stimulation (glucose, K^+^)	An amount of 20.6 mM–5.6 mM glucose	NGF secretion rises with metabolic stimulation, supporting an autocrine/paracrine role in β-cell biology and insulin regulation	N/A
[91]	Cdk5 mediates impaired autophagy by regulating NGF/Sirt1 axis to cause diabetic islet β-cell damage	Human pancreatic tissue from T2DM vs. non-diabetic patients; *db/db* and db/m mice; MIN6 mouse islet β cells under high glucose	Preclinical study	High glucose → ↑ *Cdk5* → ↓ NGF → ↓ Sirt1 → impaired autophagy → β-cell injury. NGF overexpression restores Sirt1 and autophagy; NGF blockade negates benefit of *Cdk5* knockdown, placing NGF/Sirt1 downstream of *Cdk5*	Lv-*Cdk5* shRNA (*Cdk5* knockdown) via tail vein in *db/db* mice; *Cdk5* inhibitor roscovitine; NGF siRNA; NGF overexpression plasmid; NGF inhibitor K252a in MIN6 cells	A total of 40 μL of 1 × 10^6^ IU Lv-Cdk5 shRNA; NGF siRNA 100 nM	*Cdk5* inhibition/knockdown upregulated NGF and Sirt1, normalized autophagy markers, reduced β-cell injury; K252a attenuated these protective effects, confirming *Cdk5* → NGF/Sirt1 → autophagy as a causal axis relevant to diabetic β-cell damage	N/A
[92]	NGF-withdrawal induces apoptosis in pancreatic β cells in vitro	Primary human pancreatic islets, purified human β cells, mouse β-cell line βTC6-F7	Preclinical study	β-cells express NGF and TrkA/p75NTR; NGF withdrawal triggers apoptosis via TrkA (↓ PI3K/AKT/Bad survival; ↑ JNK)	Neutralizing anti-NGF mAb; TrkA inhibition (Tyrphostin AG879); transcription/translation blockade controls	Anti-NGF 10 µg/mL; AG879 50 µM; cycloheximide 1 µg/mL; actinomycin D 1 µg/mL	Integrity of the NGF/TrkA axis supports β-cell survival; NGF removal is sufficient to induce apoptosis independent of de novo transcription/translation	N/A
[88]	Neurotrophin Signaling Is Required for Glucose-Induced Insulin Secretion	Mouse/human islets; pancreatic β-cells	Preclinical study	Vascular-to-β cell NGF–TrkA paracrine axis is essential for GSIS. NGF is produced by pancreatic vascular contractile cells; TrkA is localized on β cells. High glucose rapidly increases NGF secretion and TrkA phosphorylation; vascular NGF or β cell TrkA loss, or acute TrkA inhibition, impairs GSIS, glucose tolerance, F-actin remodeling, and insulin granule exocytosis. Exogenous NGF potentiates GSIS in human islets, establishing NGF–TrkA signaling as a critical regulator of β-cell function.	β cell–specific TrkA deletion (*Pdx1*-Cre;TrkA f/f); vascular NGF deletion (*Myh11*-CreERT2;NGF f/f); acute TrkA inhibition with 1NMPP1 in TrkA F592A mice and isolated islets; recombinant NGF added to mouse and human islets; adenoviral expression of TrkA/TrkA mutants and pharmacologic modulators of TrkA endocytosis	NGF (100 ng/mL, 30 min)	NGF–TrkA signaling, including TrkA endocytosis and actin remodeling, is required for normal glucose-induced insulin secretion; vascular NGF and β-cell TrkA are necessary for glucose tolerance, and NGF augments GSIS in human islets → NGF–TrkA axis	N/A
[93]	Nerve growth factor increases in pancreatic beta cells after streptozotocin-induced damage in rats	STZ-induced diabetes rats	Preclinical study	Streptozotocin administration induced β-cell injury with a marked reduction in insulin secretion, accompanied by increased NGF expression and release, suggesting an early endogenous protective response.	Direct exposure of isolated β-cells (in vitro)		NGF upregulation represents an early autocrine defense against cytotoxic stress in β-cells; insufficient to prevent cell loss and apoptosis	Not reported
[94]	Heterogeneous enhancer states orchestrate β-cell responses to metabolic stress	Mouse islets from normal chow vs. HFD-fed mice profiled by single-nucleus RNA + H3K27ac/H3K4me1 multiome; MIN6 β-cells; *Ins2* WT/C96Y Akita diabetic mice	Preclinical study	Multi-omic (snRNA-seq + epigenomics) mapping with computational ligand–receptor inference, followed by in vitro and in vivo functional validation	Multi-omic and cell–cell communication analyses identify NGF as a fibroblast/ductal-derived paracrine ligand with high regulatory potential on ER-stress genes in β cells. NGF expression is enriched in non-β islet cells; TrkA is expressed on β cells. Recombinant NGF suppresses ER stress/UPR markers (*Atf3*, *Ddit3*/CHOP, *Hspa5*, etc.) in HFD islets and TG-stressed MIN6 cells in a TrkA-dependent manner, indicating that NGF–TrkA signaling directly protects β cells from maladaptive ER stress. In Akita mice, systemic NGF ameliorates ER-stress–driven β-cell failure, linking NGF signaling to preserved insulin content and improved glycemic control.	Recombinant mouse NGF; pretreated with NGF.	NGF 100 ng/mL (3 h pretreatment); NGF 1 mg/kg i.p. once daily for 2 weeks in Akita mice.	N/A
[95]	Immunohistochemical examination of cinnamon extract on NGF and TrkA distribution in pancreatic tissue of diabetic rats	STZ-induced diabetic rats	Preclinical study	Cinnamon extract increased NGF and TrkA expression in pancreatic β-cells of diabetic rats	Cinnamon extract by oral gavage	A total of 200 mg/kg	Improved pancreatic histology and enhanced NGF/TrkA expression vs. untreated diabetics	N/A
[96]	Nerve growth factor is associated with islet graft failure following intraportal transplantation	STZ-induced diabetic BALB/c mice; syngeneic islet transplantation into portal vein	Preclinical study	Ex vivo, pre-incubation of islets with NGF increased apoptosis and impaired revascularization, reducing post-transplant engraftment	Pretransplant islet culture with mouse NGF followed by intraportal injection of syngeneic islets into STZ-diabetic recipients	NGF 0, 20, 100, 500 ng/mL for 24 h; transplantation arm: islets cultured 24 h with 100 ng/mL NGF before intraportal infusion; STZ 200 mg/kg i.p. to induce diabetes	Reduced engraftment with increased apoptosis and impaired revascularization after NGF exposure	N/A
[97]	Hyperglycemic tumor microenvironment induces perineural invasion in pancreatic cancer	Pancreatic cancer cell lines; DRG/Schwann cell co-cultures; diabetic/normal nude mice bearing PanCa xenografts	Preclinical study	Hyperglycemia upregulated NGF and TrkA/p75NTR in PanCa cells; NGF neutralization reduced proliferation/invasion; hyperglycemia aggravated PNI in vivo	NGF-neutralizing antibody in cell assays and xenografts	Antibody and inhibitor dosing per Methods	Blocking NGF/TrkA signaling mitigated PNI and tumor–nerve interactions driven by hyperglycemia	Not reported

Abbreviations: Ab, antibody; AE, adverse events; DRG, Dorsal Root Ganglion; ER, Endoplasmic Reticulum; GSIS, Glucose-Stimulated Insulin Secretion; HFD, High Fat Diet; i.p., intraperitoneal; N/A, not available; NGF, Nerve Growth Factor; PanCa, Pancreatic Cancer; STZ, Streptozotocin; TG, thapsigargin; UPR, Unfolded Protein Response. Symbols: ↑, increased levels/expression; ↓, decreased levels/expression; → direction of effect/sequence.

**Table 7:** Clinical studies investigating NGF signaling in humans.

Ref.	Title	Diabetic Complication	Model/ Population	Design/Type	NGF/proNGF Role/ Biological Finding	Intervention/Administration	Dose	Outcomes/ Conclusions	AE
[98]	Efficacy and safety of recombinant human nerve growth factor in patients with diabetic polyneuropathy: A randomized controlled trial	Diabetic Peripheral Neuropathy	A total of 460 adult patients with symptomatic DPN (multicenter, USA and Europe)	Phase III randomized, double-blind, placebo-controlled clinical trial (48 weeks)	Systemic rhNGF aimed to restore neurotrophic support; no clinically meaningful improvement on composite neurological outcomes	Subcutaneous rhNGF three times weekly vs. placebo	An amount of 0.1 μg/kg s.c. (T.I.W.) for 48 weeks	No significant benefit on NIS-LL, nerve conduction or global clinical assessments	Injection-site pain/hyperalgesia frequent; higher dropout in rhNGF arm; no major systemic toxicity
[99]	Fulranumab for treatment of diabetic peripheral neuropathic pain: a randomized controlled trial	Diabetic peripheral neuropathy	A total of 77 Adults with painful DPN	Phase II randomized, double-blind, placebo-controlled trial	Anti-NGF monoclonal antibody blocks NGF-TrkA/p75 signaling to reduce nociceptor sensitization; NGF levels not measured	Subcutaneous fulranumab vs. placebo every 4 weeks	Either 1, 3, or 10 mg/4 weeks	Dose-responsive analgesic signal; 10 mg group achieved greater pain reduction and higher responder rates vs. placebo	Class-typical AEs (arthralgia, edema, diarrhea) in short-term follow-up
[100]	Nerve Growth Factor Improves the Outcome of Type 2 Diabetes–Induced Hypotestosteronemia and Erectile Dysfunction	ED and hypotestosteronemia in T2DM and sensorimotor PN	A total of 148 male T2DM patients (IIEF-5 < 21); TM3 Leydig cells (MGO-induced injury)	Randomized open-label clinical study + in-vitro mechanistic experiments	NGF improved Leydig-cell mitochondrial function and up-regulated StAR/CYP11A1 via TrkA, enhancing steroidogenesis	NGF 18 mg/day intramuscularly added to standard therapy; in vitro NGF 25 ng/mL	A total of 18 mg/day IM for 10 days; in-vitro 25 ng/mL	Greater increases in total and free testosterone and IIEF-5 vs. controls; mechanistic support from Leydig-cell assays	N/A
[101]	The Effects of Tocotrienol-Rich Vitamin E (Tocovid) on Diabetic Neuropathy: A Phase II Randomized Controlled Trial	Diabetic Peripheral Neuropathy	A total of 80 T2D adults with DPN	Phase II randomized, double-blind, placebo-controlled clinical trial (12 months)	Antioxidant therapy reduced oxidative stress and improved peripheral nerve indices; NGF modulation not directly measured	Tocotrienol-rich vitamin E vs. placebo	An amount of 200 mg b.i.d. (Tocovid) × 12 months	Improved nerve conduction velocities, symptoms and small-fiber measures vs. placebo	Well tolerated; no significant adverse events reported
[102]	Effect of rosuvastatin on diabetic polyneuropathy: a randomized, double-blind, placebo-controlled Phase IIa study	Diabetic Peripheral Neuropathy	An amount of 34 T2D patients with DPN	Randomized, double-blind, placebo-controlled Phase IIa (12 weeks)	Antioxidant/pleiotropic effects improved neuropathy indices, while circulating β-NGF remained unchanged	Rosuvastatin 20 mg daily vs. placebo	Rosuvastatin 20 mg/day orally for 12 weeks	Reduced neuropathy symptoms/disability and oxidative stress; improved nerve conduction without NGF change	Well tolerated; AE profile similar to placebo
[11]	Phase II Randomized, Double-Masked, Vehicle-Controlled Trial of Recombinant Human Nerve Growth Factor for Neurotrophic Keratitis	Neurotrophic keratopathy	Adults with stage 2–3 neurotrophic keratitis (NK); 12/156 (7.7%) had diabetes or mixed diabetic etiology	Multicenter, randomized (1:1:1), double-masked, vehicle-controlled phase II trial; 8-week treatment + 48–56-week follow-up	Topical NGF supports corneal epithelial healing and sensory nerve regeneration through local trophic activity	Topical rhNGF eye drops 6×/day vs. vehicle	A total of 10 μg/mL and 20 μg/mL solutions for 8 weeks	Week-8 complete healing 74–75% (rhNGF) vs. 43% (vehicle); durable benefit at follow-up	Well tolerated; mainly mild transient ocular events; no systemic toxicity
[103]	Topical application of nerve growth factor in human diabetic foot ulcers: A study of three cases	Diabetic foot ulcers	Human; three patients with chronic diabetic foot ulcers	Clinical case series	Topical NGF provided local trophic support, promoting angiogenesis, re-epithelialization, and nerve fiber sprouting at wound edge	Topical NGF solution applied directly on ulcer surface	A total of 0.5 mg NGF diluted in 10 mL 0.9% NaCl; applied with dressings	Accelerated epithelial closure; complete healing within 8–12 weeks; reduced pain; improved local innervation	Not reported

Abbreviations: AE, adverse events; b.i.d., bis in die (twice daily); DPN, Diabetic Peripheral Neuropathy; ED, Erectile Dysfunction; IIEF-5, International Index of Erectile Function—five items; IM, Intramuscular; MGO, Methylglyoxal; N/A, not available; NIS-LL, Neuropathy Impairment Score—Lower Limbs; NK, Neurotrophic Keratopathy; PN, Peripheral Neuropathy; rhNGF, recombinant human nerve growth factor; s.c., subcutaneous; StAR, Steroidogenic Acute Regulatory Protein; T2DM, Type 2 Diabetes Mellitus; T.I.W., ter in week.

## Data Availability

No new data were created or analyzed in this study. Data sharing is not applicable to this article.

## Abbreviations

The following abbreviations are used in this manuscript:

AGEs	Advanced Glycation End Products
AMPK	AMP-Activated Protein Kinase
BDNF	Brain-Derived Neurotrophic Factor
β-FGF	Beta Fibroblast Growth Factor
CGRP	Calcitonin Gene-Related Peptide
CHOP	C/EBP Homologous Protein
DBD	Diabetic Bladder Dysfunction
DCM	Diabetic Cardiomyopathy
DE	Diabetic Encephalopathy
DN	Diabetic Neuropathy
DPN	Diabetic Peripheral Neuropathy
DR	Diabetic Retinopathy
DRG	Dorsal Root Ganglia
DPP-4	Dipeptidyl Peptidase-4
ER	Endoplasmic Reticulum
GDNF	Glial Cell Line-Derived Neurotrophic Factor
GSIS	Glucose-Stimulated Insulin Secretion
HbA1c	Hemoglobin A1c
hAFSC	Human Amniotic Fluid Stem Cells
HO-1	Heme Oxygenase-1
I/R	Ischemia–Reperfusion
MAPK	Mitogen-Activated Protein Kinase
mNGF	mature Nerve Growth Factor
N-CAM	Neural Cell Adhesion Molecule
NF-κB	Nuclear Factor kappa-light-chain-enhancer of activated B cells
NGF	Nerve Growth Factor
OAB	Overactive Bladder
PI3K	Phosphoinositide 3-Kinase
proNGF	precursor Nerve Growth Factor
p75NTR	p75 Neurotrophin Receptor
RAGE	Receptor for Advanced Glycation End Products
RGCs	Retinal Ganglion Cells
rhNGF	recombinant human Nerve Growth Factor
SP	Substance P
STZ	Streptozotocin
T2DM	Type 2 Diabetes Mellitus
TNF-α	Tumor Necrosis Factor alpha
TrkA	Tropomyosin receptor kinase A
TRPV1	Transient Receptor Potential Vanilloid-1
VEGF	Vascular Endothelial Growth Factor
